# Intron retention-dependent gene regulation in *Cryptococcus neoformans*

**DOI:** 10.1038/srep32252

**Published:** 2016-08-31

**Authors:** Sara Gonzalez-Hilarion, Damien Paulet, Kyung-Tae Lee, Chung-Chau Hon, Pierre Lechat, Estelle Mogensen, Frédérique Moyrand, Caroline Proux, Rony Barboux, Giovanni Bussotti, Jungwook Hwang, Jean-Yves Coppée, Yong-Sun Bahn, Guilhem Janbon

**Affiliations:** 1 Institut Pasteur, Unité Biologie des ARN des Pathogènes Fongiques, Département de Mycologie, F-75015, Paris, France; 2 Institut Pasteur, Plate-forme transcriptome et Epigénome, Département Génomes et Génétique, F-75015, Paris, France; 3 Department of Biotechnology, College of Life and Biotechnology, Yonsei University, Seoul 03722, Korea; 4 RIKEN Center for Life Science Technologies, Yokohama Institute, Division of Genomic Technology, Yokohama, 230-0045, Japan; 5 Institut Pasteur, HUB Bioinformatique et Biostatistique, C3BI, USR 3756 IP CNRS, F-75015, Paris, France; 6 Graduate School of Biomedical Science and Engineering, Hanyang University, Seoul, Korea

## Abstract

The biological impact of alternative splicing is poorly understood in fungi, although recent studies have shown that these microorganisms are usually intron-rich. In this study, we re-annotated the genome of *C. neoformans* var. *neoformans* using RNA-Seq data. Comparison with *C. neoformans* var. *grubii* revealed that more than 99% of ORF-introns are in the same exact position in the two varieties whereas UTR-introns are much less evolutionary conserved. We also confirmed that alternative splicing is very common in *C. neoformans*, affecting nearly all expressed genes. We also observed specific regulation of alternative splicing by environmental cues in this yeast. However, alternative splicing does not appear to be an efficient method to diversify the *C. neoformans* proteome. Instead, our data suggest the existence of an intron retention-dependent mechanism of gene expression regulation that is not dependent on NMD. This regulatory process represents an additional layer of gene expression regulation in fungi and provides a mechanism to tune gene expression levels in response to any environmental modification.

Alternative splicing increases diversity in transcriptome and proteome profiles by generating multiple mRNA isoforms from a single gene^1^. Three main types of alternative splicing have been described: exon skipping (ES), alternative splice site selection (A-SS), and intron retention (IR)^2^. In metazoans, in which most genes contain introns and the vast majority are alternatively spliced, it is well documented that alternative splicing can have a great impact on gene function, tissue-specific expression, and disease^3^,^4^,^5^. In fungi, the biological impact of alternative splicing has been poorly studied in part because most research has been done in *Saccharomyces cerevisiae*, an organism with very few introns (<5% of genes contain introns). Nevertheless, in this model yeast the intron containing genes represent 50% of the total transcripts because they are highly expressed genes and recent analyses of next generation sequencing data revealed numerous cases of alternative splicing^6^,^7^. In addition, in recent years a large number of fungal genomes have been sequenced and introns appear to be more common than previously anticipated. For example, the percentage of intron-containing genes ranges from 2.4% in *Candida glabrata*, 14.5% in *Yarrowia lipolytica*, and 47% in *Schizosaccharomyces pombe* to >99% in *Cryptococcus neoformans*^8^,^9^,^10^. Moreover, recent reports suggest that alternative splicing is not rare in fungi^11^,^12^. These studies revealed that different types of alternative splicing occur in varying proportions among the kingdoms. In fungi and plants, IR is the most common type of alternative splicing and ES is very rare, while ES is the prevalent type of alternative splicing in animals^11^,^12^,^13^,^14^. Some studies also suggest that alternative splicing plays a role in the regulation of virulence in pathogenic fungi^13^,^15^,^16^,^17^, though few cases of functional alternative splicing have been reported^18^. At this time, the biological functions of alternative splicing in fungi remain largely unknown.

The encapsulated basidiomycete pathogenic yeast *C. neoformans* is an intron-rich organism. *C. neoformans* exists as two varieties (var. *grubii* and var. *neoformans*) characterized by distinct epidemiological distribution and pathobiological properties^19^. A recent re-annotation of the *C. neoformans* var. *grubii* genome revealed spectacular complexity of its transcriptome^10^. More than 99% of the genes contain introns, and most are multi-intronic with an average of 5 introns per gene. These introns have been shown to be necessary for gene expression^20^. Moreover, preliminary analyses suggested that all types of alternative splicing are present although, as in other fungi, IR appears to be most common^10^.

In this study, we re-annotated the genome of the pathogenic fungus *C. neoformans* var. *neoformans* using RNA-Seq data. This analysis revealed thousands of alternative splicing events affecting a very large number of genes in this yeast. We show that these alternative splicing events mostly result from intron retention and are tightly and specifically regulated by growth conditions. We also provide evidence demonstrating that alternative splicing does not greatly impact proteome diversity. Instead, our data suggest that intron retention regulation provides a mechanism for *C. neoformans* to tune gene expression levels in response to external cues and may aid the pathogen in survival and proliferation in diverse environmental niches.

## Results

### Intron positioning in *C. neoformans* var. *neoformans*

The previous annotation of the *C. neoformans* var. *neoformans* genome largely depended on bioinformatics sequence analysis and comparison with other organisms^21^. Although it is a reasonable strategy to obtain a first draft of a genome annotation, our previous re-annotation of the *C. neoformans* var. *grubii* genome revealled that intron and exon positions are very difficult to predict bioinformatically and that a large proportion of the predicted protein sequences might be wrong when no transcriptomic data is available^10^. Thus, to precisely localize all introns within the *C. neoformans* var. *neoformans* genome, deep-coverage paired-ends strand-specific RNA sequences were generated from 6 different conditions in triplicate. A total of 1.4 × 10^9^ strand-specific sequences were aligned to the JEC21 reference genome. Read alignments were compared to the initial gene set of 6,273 predicted coding genes. We found at least 30 reads spanning predicted exon/intron boundaries for 86% of the introns present in the annotation (n = 37,814), confirming the *in silico* predicted gene structures. In contrast, 2.3% of the annotated introns had no spanning reads despite being within an expressed gene, suggesting potential incorrect annotations. More notably, we identified 4,782 new introns, resulting in sequence alteration of nearly one-third of the coding sequences (n = 2,102). We also identified 497 new coding genes and removed 131 coding genes of the original set, mainly through gene fusion or through re-annotation as pseudogenes (n = 31) (Table S1). Overall, 6,639 protein-coding genes and 231 pseudogenes were annotated.

**Figure 1:**
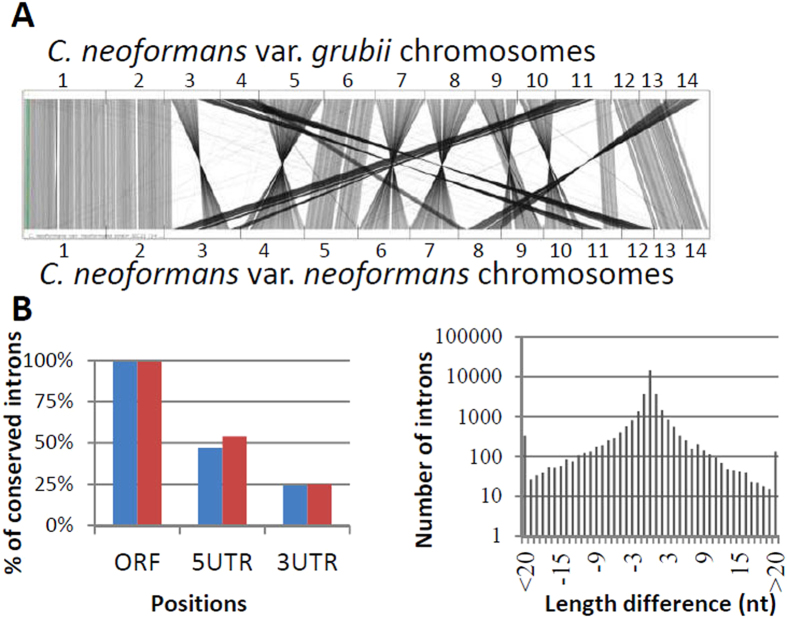
Genome comparison between *C. neoformans* var. *neoformans* and *C. neoformans* var. *grubii.* **(A)** Position of orthologous genes in *C. neoformans* var. *neoformans* and *C. neoformans* var. *grubii* as visualized using SynTView^63^. **(B)** *Right panel.* Conservation of intron position between *C. neoformans var. neoformans* and *C. neoformans* var. grubii according to the position of the introns within the genes. The blue bars represent the percentage of var. *grubii* introns conserved in the var. *neoformans* and the red bars represent the percentage of var. *neoformans* introns conserved in the var. *grubii. Left panel*. Size conservation of the ORF introns between the two varieties.

In order to validate our new annotation and to gain some insights concerning the evolutionary conservation of intron positions within the *C. neoformans* varieties, we compared the protein-coding gene structures of the var. *neoformans* with those of the var. *grubii*. First, we used a BLASTp analysis to identify 370 proteins of *C. neoformans* var. *grubii* and 112 proteins of *C. neoformans* var. *neoformans* that share no homology (*P* value > 10^−5^) to any proteins in the other variety (Table S2). This apparent discrepancy between the number of variety-specific genes might be due in part to differences in the size of telomeric and sub-telomeric regions available from the two sets, as *C. neoformans* var. *grubii* variant-specific genes map predominantly to chromosomal extremities (Figure S1). Interestingly, we also identified variety-specific transcription factors in *C. neoformans* var. *grubii* (CNAG_07370 or CNAG_03745), suggesting the existence of specific regulons. In addition, protein sequence clustering analysis^22^ (see methods) revealed the presence of 122 and 129 protein families of at least 2 members in *C. neoformans* var. *neoformans* and *C. neoformans* var. *grubii*, respectively. In both varieties, the largest variety-specific protein families were related to transposable or retrotransposable elements (Table S3). Finally, reciprocal BLAST (Bdbh) analysis identified 6,341 couples of orthologous proteins between these organisms (Fig. 1A, Table S4). To study the conservation of intron position between the two *C. neoformans* varieties, we restricted our analysis to the 5,712 couples for which the untranslated regions (UTRs) were annotated. As shown in Fig. 1B, >99% of the ORF introns are conserved in the exact same position. Nevertheless, we identified 97 ORF introns of variety *neoformans* that were lost in variety *grubii* and conversely 119 introns of variety *grubii* that were lost in variety *neoforman*s. The UTR introns appeared to be much less conserved in the 5′ UTR and even less in the 3′ UTR (Fig. 1B), suggesting that the strong evolutionary pressure that maintains the introns in the same position in the ORF is less marked for these introns. Finally, we compared intron size within the 31,555 couples of orthologous ORF introns and found that 80% differ by <2 nucleotides (Fig. 1C), confirming the high conservation of intron content between the two *C. neoformans* varieties.

### Analysis of alternative splicing

**Figure 2:**
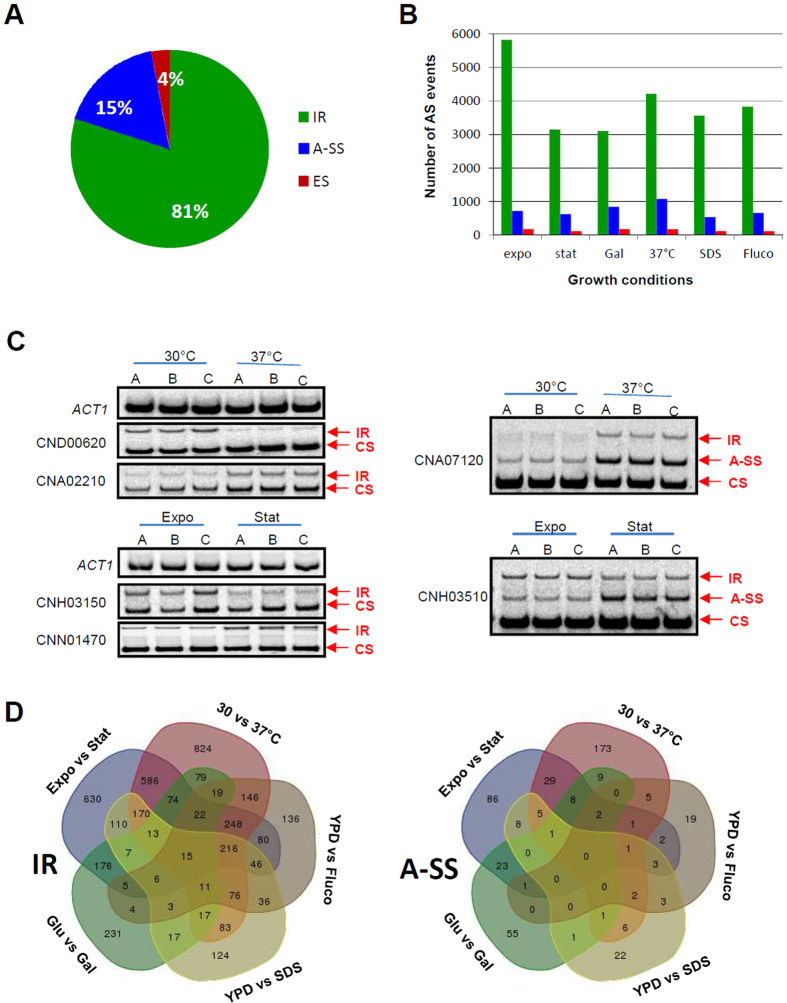
Regulation of AS by the environment. **(A)** Repartition of the different classes of alternative splicing in *C. neoformans*. **(B)** Number of AS events identified in each growth condition (i.e. exponential phase 30 °C [Expo]; exponential phase 37 °C [37 °C], stationary phase glucose [Stat D]; stationary phase galactose [Stat G]; SDS 0.01% treatment [SDS]; 10 mg /L fluconazole treatment [Fluco]). **(C)** Examples of RT-PCR analyses of alternative splicing regulation by temperature and stage of growth in *C. neoformans*. Actin mRNA was used as a control. CS, A-SS, and IR stand for constitutive splicing, alternative splice site utilization, and intron retention, respectively. Full-length blots/gels are presented in Supplementary Figure S9. **(D)** Venn diagrams drawn using lists of AS events in each variety, revealing the specificity of AS regulation by environmental cues.

We first attempted to build a catalogue of all alternative splicing events in *C. neoformans* and then studied their regulation by environmental cues. To evaluate the extent of alternative splicing in *C. neoformans*, we first defined a list of 37,814 constitutive introns (35,822 in ORF, 788 in 3′ UTR, and 1204 in 5′ UTR) in the coding genes. We then considered the three types of alternative splicing events: IR, A-SS, and ES (see Material and Methods). We identified 1462 alternative splicing events due to A-SS, associated with 1,270 constitutive introns and 7,879 cases of IR. Overall, 59% of the genes have alternatively spliced transcripts in *C. neoformans* var. *neoformans*. Consistent with previous observations in variety *grubii* and other fungi^10^,^12^,^13^, IR was the most common form of alternative splicing, followed by A-SS (Fig. 2A). Few cases of ES were observed (Fig. 2A).

Notably, the number of alternative splicing events identified varied between different environmental conditions (Fig. 2B). This finding prompted us to study potential regulation of alternative splicing by environmental cues. We found that more than half (n = 4,210) of the IR events and 32% (n = 466) of A-SS events were regulated by the growth conditions (as determined by ≥1.5-fold change) (Table S5). Semi-quantitative RT-PCR experiments were performed to confirm selected examples of this regulation. Examples of IR and A-SS regulated by the temperature and the growth stage are presented in Fig. 2C. Moreover, the results obtained for the gene CNH03510 illustrated that A-SS and IR at the same locus were not always co-regulated. Interestingly, comparison of the lists of the alternative splicing events regulated in response to a change in one parameter of the growth conditions revealed that many of these regulation changes were specific to the modification (change in temperature, carbon source, or growth stage, or the addition of SDS or fluconazole). Indeed, as shown in Fig. 2D, 46% and 76% of the IR and A-SS regulation, respectively, are comparison specific, suggesting specific alternative splicing regulations in response to environmental modification.

**Figure 3:**
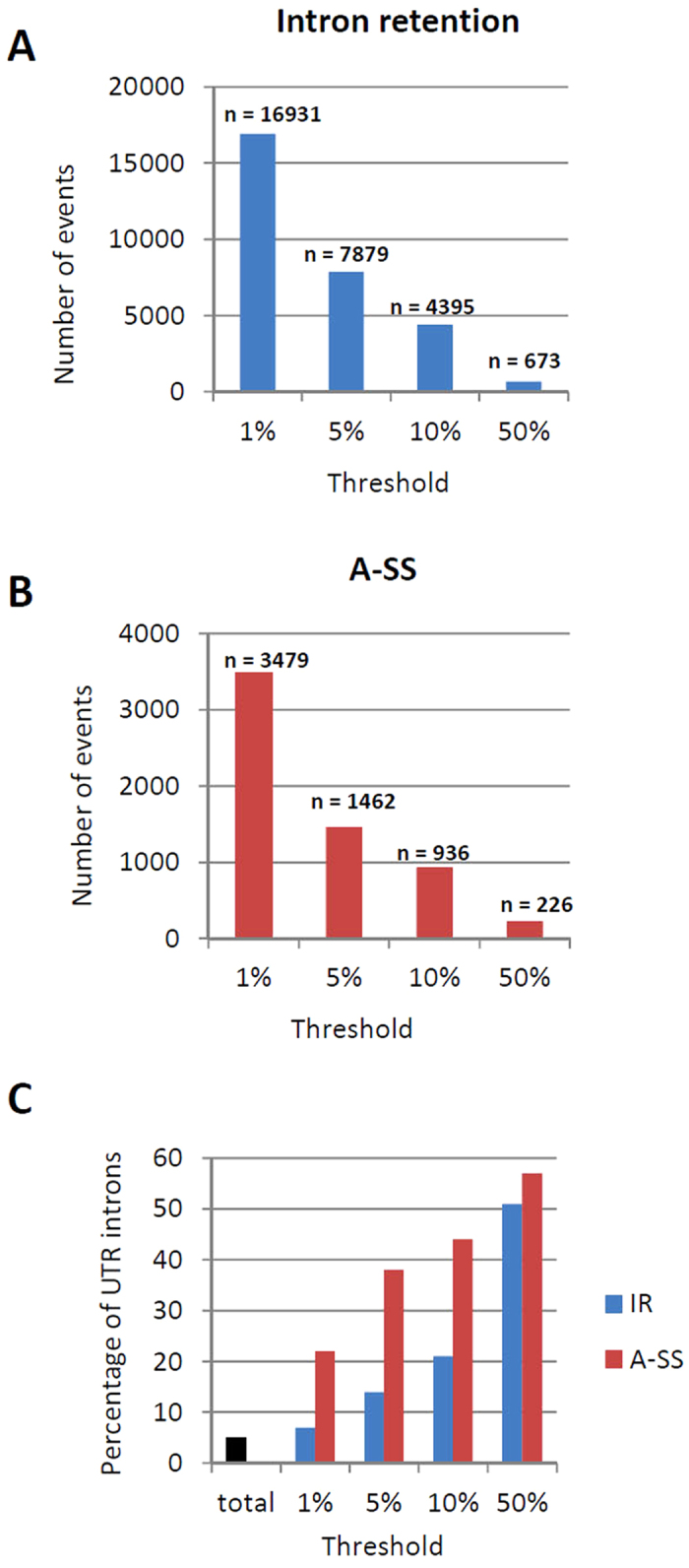
Alternative splicing contributes little to proteome diversity in *C. neoformans*. **(A)** Relationship between the number of IR events identified and the threshold used for identification. **(B)** Relationship between the number of A-SS events identified and the threshold used for identification. **(C)** Relationship between the number of AS events identified in the UTRs and the threshold used for identification.

Although IR affects a large number of genes in *C. neoformans*, several lines of evidence suggest that this contributes little to proteomic diversity. First, the mRNAs bearing a retained intron represent only a small fraction of the major type of mRNA of a single gene. As explained in the Material and Methods, an intron was considered to be regulated by IR when at least 5% of the polyadenylated transcripts retained this intron. However, modifying the threshold dramatically altered the number of events. For instance, 4,395 introns were retained in at least 10% of the transcripts in at least one condition, whereas 16,931 introns are retained in at least 1% of the transcripts in at least one condition (Fig. 3A). It is important to note here that these modifications of the threshold only poorly affect the percentages of alternative splicing regulated neither the observed specificity of these regulations (Figure S2). Second, UTR-introns are more likely to be retained than ORF-introns. Thus, the proportion of UTR-introns regulated by IR increases from 7% to 51% when the threshold for the identification of an IR event was modified (Fig. 3C). Third, most IR events are associated with the synthesis of a premature termination codon (PTC)-containing mRNA. Thus, only 31.8% of ORF introns were in-frame with the coding sequence, and 80.1% of these in-frame introns contained a stop codon. Overall, only 7% of the IR events can theoretically result in the production of an alternative protein, underlining the limited potential of IR to regulate proteome diversity in *C. neoformans*. The same is true for alternative splicing events due to 5′ and 3′ A-SS. The number of A-SS events dramatically decreased when the identification threshold increased, whereas the proportion of events identified in the UTR increased (Fig. 3B,C). Finally, 71% of the A-SS splicing events introduced a frameshift in the coding sequence. Thus, similar to IR, A-SS appears to be a very limited source of proteome diversity in *C. neoformans,* even though some of these frameshifts could result in the production of shorter proteins.

### IR level is mostly independent of nonsense-mediated mRNA decay

Most alternative splicing events in the ORF likely introduce a PTC into the mRNA sequence. These aberrant mRNAs are expected to be targets of mRNA quality control pathways, thereby avoiding the synthesis of potentially harmful truncated proteins^23^. Among these, the best studied surveillance pathway is the nonsense-mediated mRNA decay (NMD) pathway, which upon translation recognizes and degrades mRNAs that bear a PTC as a consequence of either a nonsense mutation or a non-productive splicing event^24^,^25^,^26^,^27^. Thus, we expected most of the alternative mRNA molecules in *C. neoformans* to be potential NMD targets.

Upf1, Upf2, and Upf3 proteins form the core machinery of the NMD pathway in all eukaryotes studied to date^24^. As expected, we identified a homolog for each *UPF* gene in the genomes of both *C. neoformans* varieties (*UPF1* [loci CNC02960 and CNAG_01807], *UPF2* [loci CNF01510 and CNAG_05829], and *UPF3* [loci CNG03015 and CNAG_03276]). As shown on the Figure S3A, the *upf* mutant strains did not display obvious growth phenotypes when grown at 30 °C on rich medium. The only clear phenotype observed in all of the *upf*Δ mutants of both varieties was an increased sensitivity to fluconazole. In addition, *upf* mutants in a *C. neoformans* var. *neoformans* background, but not in a *C. neoformans* var. *grubii* background, were temperature-sensitive. Finally, we found that the individual components of the NMD pathway were not required for virulence of *C. neoformans*, as each of the *upf1*∆, *upf2*∆, and *upf3*Δ individual or combined mutations resulted in no significant changes in survival patterns in a heterologous host model of cryptococcosis (Figure S3B). In conclusion, as in *S. cerevisiae* and *S. pombe,* depletion of the Upf proteins in *C. neoformans* does not affect cell viability, demonstrating that the NMD pathway is not essential in this yeast.

To get insight into the transcriptomic consequences of *UPF1* deletion, we generated strand-specific RNA-Seq data from RNA samples isolated from a wild-type and the *upf1*Δ mutant strain, both grown to the exponential phase (5 × 10^7 ^cell/mL). The paired-end, strand-specific, 100-bp reads were mapped to the *C. neoformans* var. *neoformans* genome, and statistical analysis identified genes that were significantly up-regulated at least 2-fold (n = 411) or down-regulated at least 2-fold (n = 338) in the *upf1*Δ mutant strain, respectively (Table S6). The result is very similar to those previously obtained in mammals, plants, and yeasts, in which about 1% to 10% of the genes were up-regulated upon mutation of the *UPF1* homolog^28^,^29^,^30^,^31^,^32^. Interestingly, although some of the genes, such as *CPA1* that were previously identified to be up-regulated upon NMD pathway mutation in *S. cerevisiae*^33^, were also up-regulated in *C. neoformans*, the overlap between the two species was quite poor. Thus, 157 of the 411 up-regulated genes upon *UPF1* deletion in *C. neoformans* have an ortholog in *S. cerevisiae,* but only 15 of them are up-regulated in the baker’s yeast *nam7*Δ mutant strain^34^. Moreover, GO-term enrichment analysis of the up-regulated genes did not reveal any pathway particularly altered in the *upf1*Δ mutant. More specifically, our analysis did not reveal any enrichment of genes involved in amino acid metabolism or in telomere maintenance as previously observed in other organisms^35^. In contrast GO-term analysis of the list of genes down-regulated in the *upf1*Δ strains revealed a significant enrichment of genes involved in transport. Notably, some genes like *AMF1* (encoding a homolog of a *S. cerevisiae* putative multidrug resistance transporter) and *FLR1* (encoding a homolog of a *S. cerevisiae* fluconazole transporter) were down-regulated 3.6-fold and 4.7-fold, respectively, in the *upf1*Δ mutant strain^36^. It is tempting to speculate that the down-regulation of these genes could be at least in part responsible for the fluconazole sensitivity of the NMD mutant strains. Additional experiments are needed to confirm this hypothesis.

**Figure 4:**
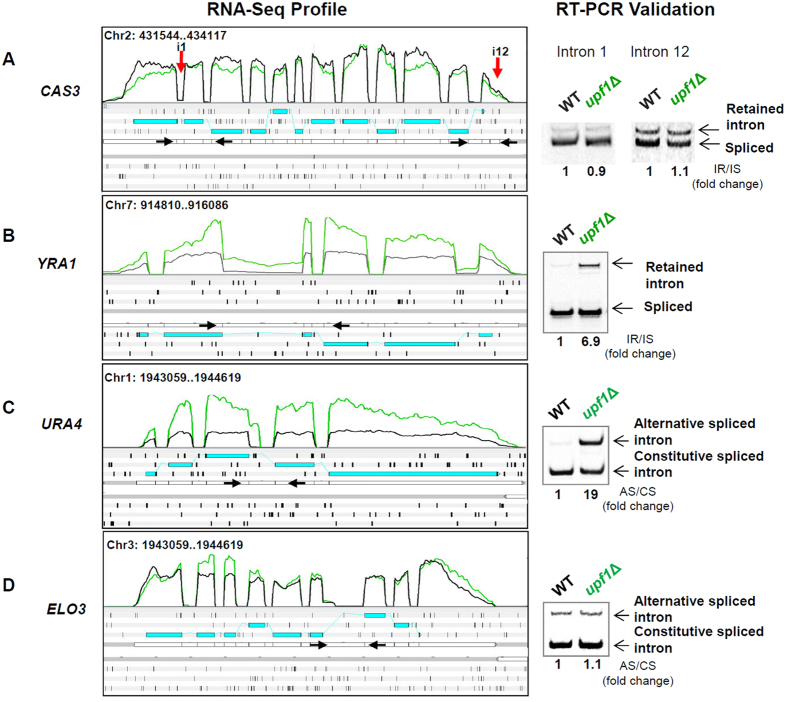
RNA-Seq profiles (left) and semi-quantitative RT-PCR validation (right) of representative alternative splicing events that are subject to or immune to NMD. The green and black curves represent the transcription coverage in the *upf1*Δ and wild-type strains, respectively; the blue boxes represent the CDS; and the vertical black bars represent the stop codons found in each frame. Primers (arrows) were chosen on the exons flanking the alternatively spliced introns in order to amplify both the constitutively spliced (CS) and the alternatively spliced (AS) or the unspliced (IR) isoform of each transcript. (**A**) *CAS3* (CNB01440) is an example of the absence of the IR regulation of upon *UPF1* deletion in *C. neoformans*. The first and the last intron were partly retained (red arrows), and this retention was not altered in the *upf1*Δ strain. (**B**) *YRA1* (CNG03240) is an example of an intron-retaining mRNA up-regulated by *UPF1* deletion. (**C**) In *URA4* (CNA07120) the use of an in-frame alternative 5′ splice site introduces a PTC on the mRNA, which is regulated in the *upf1*Δ mutant. (**D**) In the *ELO3* mRNA (CNC00110), the use of an in-frame alternative 5′ splice site does not introduce a PTC on the mRNA, and it is equally expressed in the *upf1*Δ mutant as compared to the wild-type strain. Full-length blots/gels are presented in Supplementary Figure S9.

As described above, we expected most intron-retaining mRNAs to be potential NMD targets. One might expect up-regulation of IR upon *UPF1* deletion, as has been shown for other lower eukaryotes, such as *Paramecium tetraurelia*^37^ and *S. cerevisiae*^38^,^39^. We thus studied the influence of NMD pathway deletion on IR level. Here we limited our analysis to the 5,415 introns for which IR could be measured in both wild-type replicates. Strikingly, 98% of the IR events in the ORF and 99% in the UTR were not up-regulated upon *UPF1* deletion, indicating that the vast majority of intron-retaining transcripts are not sensitive to NMD in *C. neoformans*. For instance, we previously reported that the first and last intron of the *CAS3* gene are retained in some of the polyadenylated transcripts, whereas the others are mostly efficiently spliced^20^. The present RNA-Seq-based analysis confirmed that in the wild-type strain, 5.9% and 52.9% of the *CAS3* transcripts retained introns 1 and 12, respectively, whereas none of the other introns were retained at a significant level. However, these IR levels were not affected by the deletion of *UPF1*. Indeed, these levels remained very close to those measured in the wild-type strain (6.1% and 53.9% for introns 1 and 12, respectively). These results were confirmed using semi-quantitative RT-PCR assays as shown in Fig. 4A. We also tested additional loci (CNG00160, CNK02010, and CNA04020) using semi-quantitative RT-PCR assays and obtained similar results, suggesting that NMD has little role in the control of these intron-containing RNA molecules (Figure S4).

Nevertheless, we identified 98 introns in ORFs for which retention was up-regulated in the *upf1*Δ strains (Table S7). We confirmed the effect of the *UPF1* mutation for some IR events using semi-quantitative RT-PCR. An example of the results obtained for the *YRA1* gene (locus CNG03240) is presented in Fig. 4B. We obtained similar results with the other *upf* mutant strains for both varieties (Figure S5). However, we failed to identify any specific features potentially useful to predict whether the rate of the IR event would be NMD-controlled. Thus, neither the intron size nor the intron position within the gene was predictive of an IR event coupled to NMD-dependent degradation.

In contrast to IR, 52% of the A-SS events identified in the wildtype and which potentially introduce of frameshift in the CDS were shown to be up-regulated in the *upf1∆* mutant. More interestingly, 87% (n = 297) of A-SS events up-regulated in the *upf1*Δ mutant strain introduced a frameshift in the coding sequence (Table S7). An example of this category of endogenous NMD target is depicted in Fig. 4C. The *UPF1* deletion revealed the presence of an alternative splicing event of the third intron of *URA4* (CNA07120). RT-PCR experiments using adapted primers confirmed this result. In contrast, most (77%) of the alternative splicing events that did not introduce a frameshift in the coding sequence were not up-regulated in the *upf1*Δ strain (see representative locus in Fig. 4D). Interestingly, visual examination of the remaining 23% revealed that 31 out of 30 introduced a stop codon to the coding sequence. These data suggest that NMD largely controls the consequences of non-productive A-SS events in *C. neoformans*.

### Link between IR and gene expression regulation

**Figure 5:**
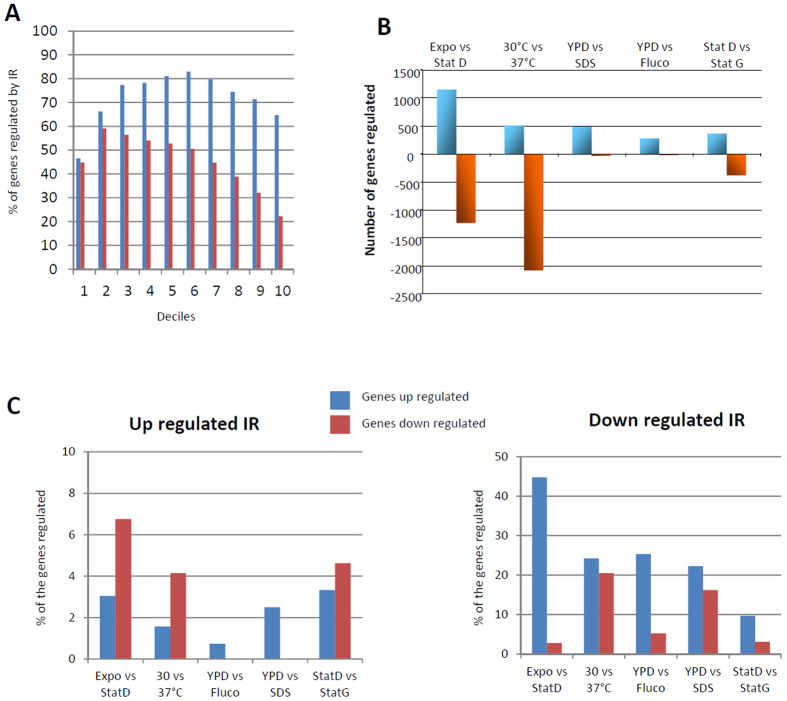
Intron retention and gene expression regulation is linked. **(A)** The most highly expressed genes are less regulated by IR than moderately expressed genes. Genes were separated into deciles according to level of expression at 30 °C in the exponential phase. The percentage of genes regulated by IR in this condition (1% threshold blue; 5% threshold red) is reported. **(B)** Gene expression regulation in each comparison. (i.e. Exponential phase 30 °C [Expo]; Exponential phase 37 °C [37 °C], Stationary phase glucose [StatD]; Stationary phase galactose [StatG]; SDS 0.01% treatment [SDS]; 10 mg/L fluconazole treatment [Fluco]).**(C)** Proportion of genes up- or down-regulated when IR is up- or down-regulated. For each comparison, the gene lists for which IR down- or up-regulation was observed were compared with the gene lists for which up- or down-regulation of expression was observed. The percentages of overlap between these lists are reported.

We analyzed the association between rate of IR and the up- or down-regulation of gene expression. For each growth condition, we arbitrary split the genes in 10 groups, and within the groups the genes were ranked from the lowest to highest level of expression. After eliminating genes that were not expressed in the studied condition (fpkm < 1), we numbered each gene that was regulated by IR within each group. As shown in Fig. 5A, at 30 °C in YPD at exponential phase, the most highly expressed and the least expressed genes tended to be less affected by IR than the genes with moderate expression. A similar pattern was obtained for nearly all conditions (Figure S6). Although the reduced number of IR-regulated genes within the lower expression group is probably due to the criteria used to identify the IR events, the relatively small number of genes affected by IR within the most highly expressed genes suggests a negative correlation between gene expression and intron retention in *C. neoformans*.

Next, we performed a statistical analysis to identify differentially regulated genes in the various growth conditions (Table S8; Figure 5B). For instance, more than a third of the genes (n = 2593) were regulated by a change in temperature. Probably due to a general down-regulation of the metabolism together with a slower growth of the cells at 37 °C, ribosome and translation associated-genes were down-regulated at this temperature. Interestingly, some RNA metabolism-associated GO-terms (RNA binding, spliceosomal complex, RNA splicing) were also enriched in the list of the down-regulated genes at this temperature, suggesting that a complex transcriptomic alteration occurs at this temperature. In our analysis, most of the up-regulated genes at 37 °C (71%) have no GO-term annotation, although intracellular and extracellular transport-related genes appeared to be up-regulated. Again these results suggest that the response of *C. neoformans* to the host temperature is not well understood. We then studied the relationship between IR level and gene expression regulation. Accordingly, up- and down-regulation of IR in the 6 conditions considered in this study were often associated with the down- and up-regulation of gene expression, respectively (Fig. 5C). For instance, down-regulation of IR was observed for 44% of the up-regulated gene in stationary phase whereas the same observation was made in only 3% of the down-regulated genes. These data clearly suggest the existence of an interdependent relationship between gene expression regulation and IR regulation in *C. neoformans*.

## Discussion

We observed that alternative splicing is common in *C. neoformans* var. *neoformans* and that IR represents its most common manifestation, confirming previous reports in *C. neoformans* var. *grubii* and other fungi^10^,^21^. However, the level of IR does not appear to be highly influenced by NMD in *C. neoformans*. In this aspect, *C. neoformans* seems to be similar to plants, in which despite a high level of IR only a minority of intron-retaining transcripts is subject to NMD^40^,^41^. Due to low levels of IR and its small likelihood to generate alternative proteome profiles, one could consider these IR events to be noise due to inefficient intron splicing. However, we previously demonstrated that artificial elimination of the retained introns in a model gene results in enhanced gene expression showing that, at least for this gene, IR is associated with down-regulation of gene expression^20^. Moreover, our present data reveal the tight and specific regulation of the IR level by growth conditions and the clear relationship between the regulation of IR level and gene expression, suggesting a controlled and regulated mechanism.

**Figure 6:**
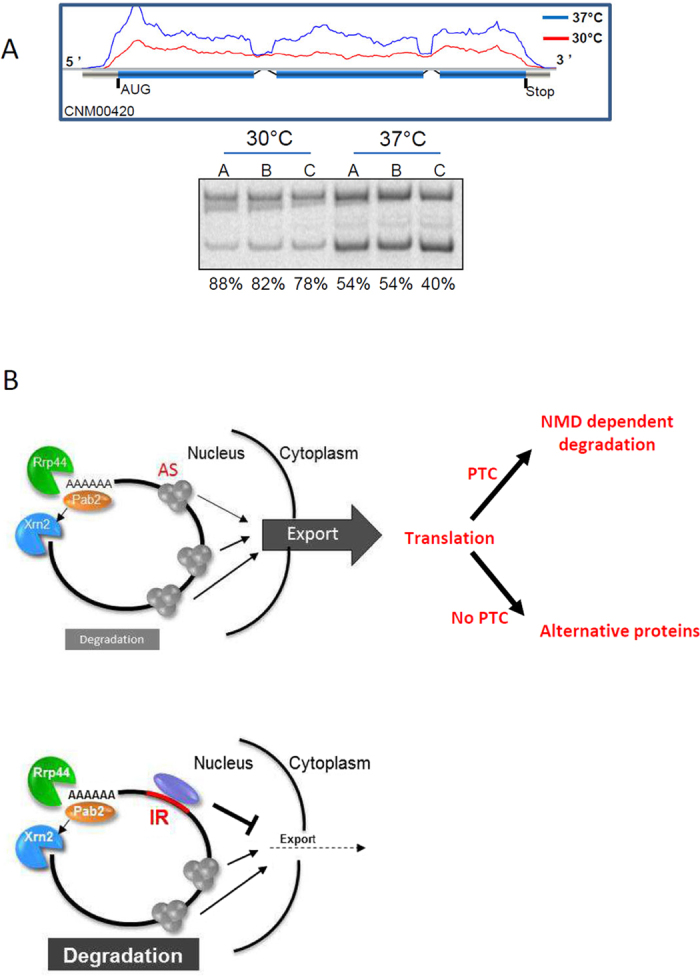
IR-dependent regulation of gene expression in *C. neoformans.* **(A)** Example of IR-dependent regulation of gene expression in gene CNM00240. *Top panel*. RNA-Seq profiles as visualized with Artemis. The blue and red curves represent transcription coverage at 30 °C and 37 °C, respectively; the blue boxes represent the CDS; and the vertical black bars represent the stop codons found in each frame. *Bottom panel*. RT-PCR analysis of intron-retention. Primers (arrows) were chosen on the exons flanking the alternatively spliced introns in order to amplify both the spliced (CS) and unspliced (IR) mRNAs. The experiment was performed on 3 independent replicates and the percentages of retention are given. Full-length blots/gels are presented in Supplementary Figure S9. **(B)** Model of alternative splicing-dependent regulation of gene expression. *Top panel*. In the case of A-SS, the mRNAs are exported to the cytoplasm. If it contains a PTC, it is degraded by the NMD pathway. In the absence of PTC an alternative protein is produced. *Bottom panel*. If an intron is retained, export from the nucleus is repressed.

Overall, our data suggest that control of gene expression by IR occurs in *C. neoformans*. Because this regulation is independent of the NMD pathway and thus probably not dependent on translation, one can imagine that this regulation takes place in the nucleus, in which incompletely spliced mRNAs would be retained. One prominent example is the regulation of IR-dependent gene expression by temperature, as shown in Fig. 6A. The CNM00420 gene was transcribed at both 30 °C and 37 °C but was poorly spliced at 30 °C. Thus, the regulation of the expression of this gene at least partly depends on temperature-dependent regulation of IR. The forces inhibiting the export of partially spliced mRNAs are unknown, but they would counteract the forces promoting the export of mRNAs upon splicing^20^. In fact, imaging experiments performed with living plant cells and subcellular fractionation experiments suggested that pre-mRNAs are retained in the nucleus in plant^42^,^43^. These intron-containing mRNAs could be discriminated from the completely spliced mRNAs by virtue of differences in the set of proteins bound to them^44^,^45^,^46^,^47^,^48^. In *C. neoformans*, a Spliceosome-Coupled And Nuclear RNAi (SCANR) complex has been shown to mediate the control of transposon expression by targeting transposon transcripts stalled on spliceosomes to degradation in the nucleus^49^. However, the strong siRNA mapping bias to transposons and to sequences sharing similarities with centromeres suggests that this mechanism is restricted to the regulation of transposon expression^49^.

Overall we propose a model (Fig. 6B) in which splicing efficiency is regulated by environmental cues. In this model, IR-regulated mRNAs are exported to the cytoplasm and/or degraded in the nucleus. Thus, IR regulation represents an additional mechanism in *C. neoformans* to finely tune the level of expression of some genes in order to efficiently adapt to diverse environments.

## Material and Methods

### Strains and culture conditions

*C. neoformans* strains used in this study originated from the serotype D strain JEC21^50^ or serotype A H99^51^ and are listed in Table S9. The strains were routinely cultured on YPD medium at 30 °C. The bacterial strain *Escherichia coli* XL1-blue (Stratagene) was used for the propagation of all plasmids.

### Construction of deletion and conditional mutants in *C. neoformans* var. *neoformans*

The *UPF1* (CNC02960), *UPF2* (CNF01510), and *UPF3* (CNBG1750) genes were deleted by biolistic transformation using a disruption cassette constructed by overlapping PCR as previously described^52^. The transformants were then screened for homologous integration as previously described. The plasmid pNAT used to amplify the *NAT* selective marker was kindly provided by Dr. Jennifer Lodge (Saint Louis University School of Medicine). The plasmid pPZP-NEO1 used to amplify the *NEO* selective marker was kindly provided by Dr. Joseph Heitman (Duke University). These cassettes were constructed using a strategy previously applied to *Neurospora crassa* deletion cassettes^53^. All primer sequences used are provided in Table S10. We constructed at least two independent strains for each gene C*. neoformans* var. *neoformans* deletion mutant and analyzed their phenotypes. For each gene, independently constructed mutants exhibited same phenotypic traits.

### Gene disruption of *UPF1*, *UPF2*, and *UPF3* in *C. neoformans* var. *grubii*

For construction of the serotype A *upf1*Δ, *upf2*Δ, and *upf3*Δ mutants, *UPF1* (CNAG_01807), *UPF2* (CNAG_05829), and *UPF3* (CNAG_03276) were disrupted in the *C. neoformans* var. *grubii* H99S strain by using the modified split marker/double joint PCR with primers listed in Table S10 as previously reported^54^. The two split gene disruption cassettes were introduced into the H99S strain by biolistic transformation as previously described^55^. For construction of the *upf1*Δ *upf2*Δ double mutant, the *UPF2* gene was disrupted in the *upf1*Δ mutant with the *NEO* selection marker. For construction of the *upf2*Δ *upf3*Δ and *upf1*Δ *upf3*Δ double mutants and the *upf1*Δ *upf2*Δ *upf3*Δ triple mutant, *UPF3* was disrupted in the *upf1*Δ, *upf2*Δ or *upf1*Δ *upf2*Δ mutants with the hygromycin B-resistant marker (*HYG*). The correct genotype of each mutant was verified by Southern blot analysis using a gene-specific probe amplified with the L1/PO primer pair listed in Table S10. We constructed at least two independent strains for each gene C*. neoformans* var. *grubii* deletion mutant and analyzed their phenotypes. For each gene, independently constructed mutants exhibited same phenotypic traits.

### RNA extraction and sequencing

Total RNA was extracted from *C. neoformans* var. *neoformans* cells grown under various conditions using a previously described protocol^56^. We performed each extraction experiment in independent triplicates for the re-annotation of the genome and the alternative splicing analysis in wild-type strain (JEC21). For the comparison between the wild-type (JEC21) and the *upf1∆* mutant strain (NE579), we performed each extraction experiment in independent duplicates. For high-throughput sequencing, strand-specific, paired-end cDNA libraries were prepared from 10 μg of total RNA using the Illumina mRNA-Seq-Sample Prep Kit according to manufacturer’s instructions. cDNA fragments of ~400 bp were purified from each library and confirmed for quality by Bioanalyzer (Agilent). Then, 100 bp were sequenced from both ends using an Illumina HiSeq2000 instrument according to the manufacturer’s instructions (Illumina). Reads were mapped to the genome of strain JEC21 as pairs with Tophat2^57^ using the “b2-sensitive” mode with minimum intron length of 5 nucleotides and default settings for other parameters. These alignments were used to correct the *C. neoformans* var. *neoformans* gene structures as previously described^10^.

The number of fragments mapped within the exons of coding genes was counted using the “intersect” function of bedtools2 suite^58^. The average number of fragments mapped within coding gene exons in a single library was 59.1 million (see details in Table S11). Differential expression was investigated using DESeq1 v1.16^59^, DESeq2 v1.4.1^60^, and edgeR v3.6.1^61^ with default settings and false discovery rate (FDR) cutoff at 0.05. Only genes with >10 mapped fragments in at least one library were considered. A gene was considered to be significantly differentially expressed when it passed the FDR cutoff in at least 2 of the 3 methods mentioned above. The fold change output from DESeq1 was considered to be the final fold change. RNA-Seq data have been deposited in the NCBI database Bioproject PRJNA272767.

### Orthologous proteins identification

Reciprocal BLAST^62^ (Bdbh) analysis was performed using the sequences of proteins from both varieties. We defined an orthologous couple when both BLASTp *P* values were below 10^−5^. The positions of orthologous genes in *C. neoformans* var. *neoformans* and C*. neoformans* var. *grubii* were visualized using SynTView^63^. The full alignment can be obtained at http://genopole.pasteur.fr/SynTView/flash/Cryptococcus_neoformans_grubii_H99/SynWeb.html.

### Protein family identification

In order to identify protein families, we performed protein sequence clusterization using CD-HIT^22^ (parameters 60%, 60%). An exact duplication of a 62,872-bp fragment at the end of chromosome 12 inverted at the end of chromosome 8 has been previously reported by Fraser and colleagues in 2005^64^. Because this duplicated region is specific to strain JEC21 and is known to have occurred during strain manipulation in the laboratory, the 21 duplicated proteins contained in this DNA region were not considered here as constituting new protein families.

### Comparison of intron positions between *C. neoformans* var. *neoformans* and *C. neoformans* var. *grubii*

To analyze intron position conservation, the two genome sequences were aligned using Mauve 2.3.1 using default parameters (http://darlinglab.org/mauve/mauve.html). We then developed a script to use the position of each extremity of an intron in one variety to infer their theoretical positions in the other variety. We used this tool to classify introns of each strain in 1 of 4 categories: (1) conserved when both predicted positions correspond to intron extremities in the tested variety, (2) cross when the introns in each variety have at least 1 nucleotide overlap, (3) out when the predicted positions are out of the orthologous gene, and (4) lost when no intron is present at the predicted position (Figure S7).

### Analysis of alternative splicing

Different strategies were used to identify alternative splicing events depending on the type. For IR, we compared the coverage within and just upstream of each constitutive intron as done previously in *C. neoformans* var. *grubii*^10^. For transcripts for which several alternative isoforms were present in the annotation, we chose the most prevalent one when wild-type cells were grown to the exponential phase at 30 °C. Briefly, we measured the coverage in the intron and the coverage within a 2-nt window of the upstream exon (Figure S8). To identify an IR event we used the following 3 criteria: (1) coverage within the upstream exon 2-nt window needed to be at least 10 reads/nt after DESeq1 normalization (DEseq Variance Stabilized Data method); (2) coverage within the intron needed to be at least 3 reads/nt after normalization; and (3) we also limited our analysis to introns displaying at least 5% intron retention (see main text). To consider an IR event as regulated in the *upf1∆* mutant strain or by a modification of the growth condition, we used 2 additional criteria: (1) the retention level should increase or decrease by at least 1.5-fold with a *P* value < 0.05 as determined by Student t test analysis, and (2) the intron should not have been identified as regulated by A-SS. Finally, we eliminated all introns in which an alternative start site has been identified. For alternative splicing due to A-SS, we also limited our analysis to introns for which the coverage within the upstream exon 2-nt window was at least 10 reads/nt after normalization. Moreover an A-SS event needed to be identified by at least 3 reads after normalization in each replicate and represent at least 5% of the constitutive splicing event. An A-SS event was considered to be regulated when the ratio of reads recognizing the alternative intron compared to the corresponding constitutive one increased or decreased at least 1.5-fold with a *P* value < 0.05 as determined by Student t test analysis.

### RT-PCR analysis

Total RNA (5 μg) was subjected to DNAse I treatment (Roche) to eliminate contaminating genomic DNA. A total of 1 μg of the DNaseI-treated RNA was then used for reverse-transcription (RT) using the QuantiTect Reverse Transcription (Qiagen) kit. The resultant cDNAs were PCR-amplified in presence of dCTP (α^33^P) (Perkin Elmer) with the primers indicated in Table S10. PCR products were resolved on 6% polyacrylamide gel and quantified using a Typhoon 9200 imager and ImageQuant 5.2 software (Molecular dynamics).

### Wax moth killing assay

To determine the role of NMD in the virulence of *C. neoformans*, independent strains of the *upf1*Δ, *upf2*Δ, and *upf3*Δ single mutants and *upf1*Δ *upf2*Δ *upf3*Δ triple mutants were tested in a *Galleria mellonella* insect model of systemic cryptococcosis. For each group, 15 *G. mellonella* caterpillars in the final instar larval stage, ranging from 200 to 300 mg in body weight, were randomly selected within 7 days from the day of shipment (Vanderhorst, Inc., St Marys Ohio USA). The wild-type (H99S) and mutant strains were grown for 16 hours at 30 °C in YPD medium, washed 3 times with phosphate-buffered saline (PBS), and resuspended in PBS. After cell concentrations were adjusted to 10^6 ^cells/mL by hemocytometer cell counting, 4 μL (4,000 *C. neoformans* cells) were inoculated per larva through the second-to-last prolegs using a 100-μL Hamilton syringe with a 10-μL needle size. PBS was injected as a non-infection control. Caterpillars were incubated at 37 °C in petri dishes in humidified plastic containers and monitored daily after injection. Caterpillars were considered to be dead when they did not move upon touch or when they displayed a black body color. Caterpillars transforming into pupa during the experiment were censored for statistical analysis. The survival curve was illustrated by Prism 6 (GraphPad) and statistically analyzed by Log-rank (Mantel-Cox) test.

## Additional Information

**How to cite this article**: Gonzalez-Hilarion, S. *et al*. Intron retention-dependent gene regulation in *Cryptococcus neoformans. Sci. Rep.* **6**, 32252; doi: 10.1038/srep32252 (2016).

## Supplementary Material

supplemtary figures and tables

Table S1

Table S2

Table S3

Table S4

Table S5

Table S6

Table S7

Table S8

Table S9

Table S10
