# Supplementary material for: Intron retention-dependent gene regulation in Cryptococcus neoformans
Source: Sci Rep. 2016 Aug 31;6:32252. doi: 10.1038/srep32252 (PMC5006051; doi:10.1038/srep32252)

Supplementary information for the manuscript: "Intron retention-dependent gene regulation in *Cryptococcus neoformans*"

SARA GONZALEZ-HILARION[1¥], DAMIEN PAULET[2¥], KYUNG-TAE LEE[5], CHUNG-CHAU HON[3], PIERRE LECHAT[6], ESTELLE MOGENSEN[1], FRÉDÉRIQUE MOYRAND[1], CAROLINE PROUX[2], RONY BARBOUX[1], GIOVANNI BUSSOTTI[6], JUNGWOOK HWANG[4], JEAN-YVES COPPÉE[2,] YONG-SUN BAHN[5] AND GUILHEM JANBON[1*]

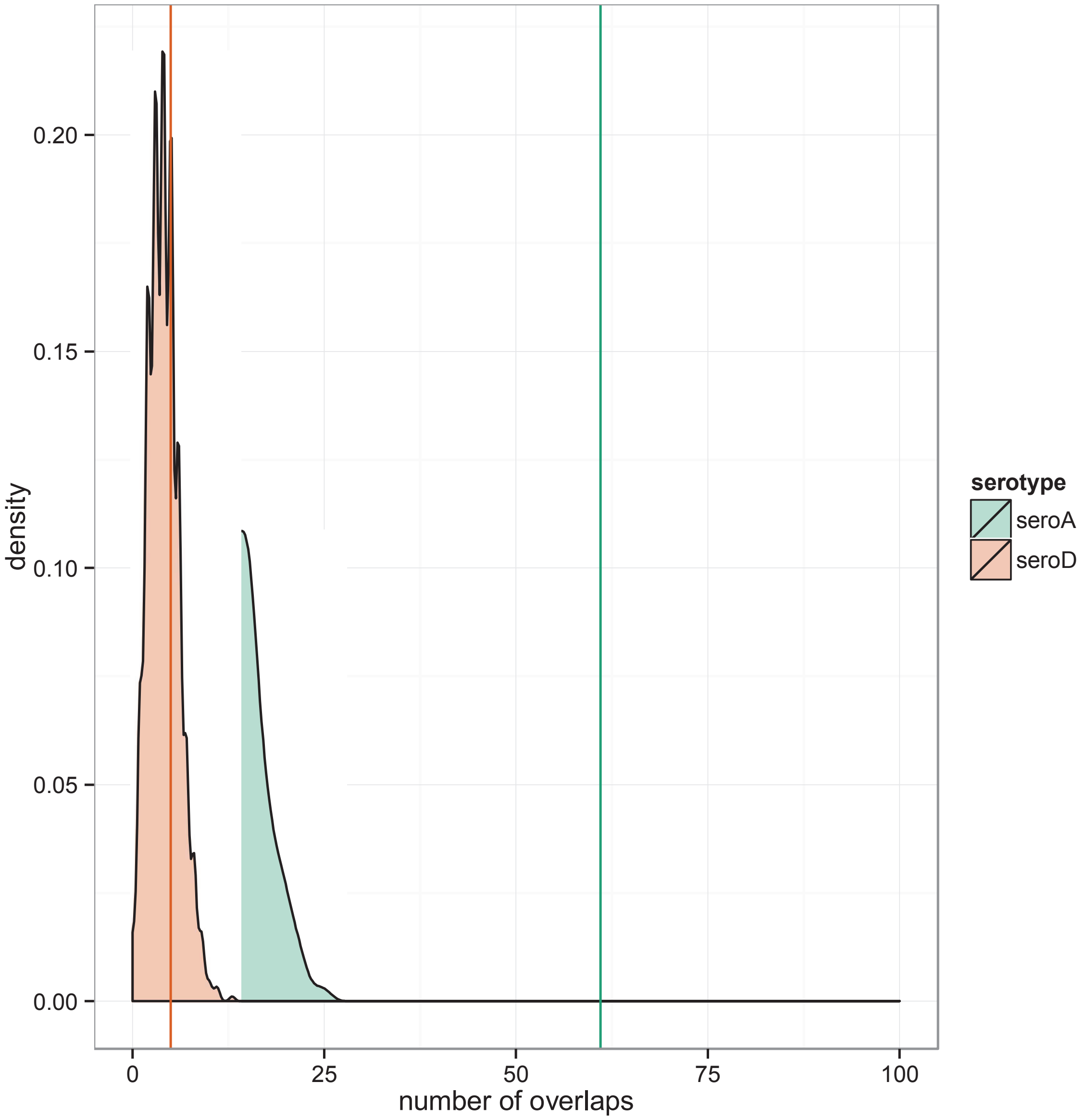

Figure S1: **Variety-specific gene position bias.** The coordinates of variety-specific genes were compared against the 25-kb terminal regions of all chromosomes. The number of overlaps between the genes and chromosome extremities is shown on the x-axis and demonstrated by vertical lines (green for var. *grubii* and orange for var. *neoformans*). To generate the density distributions (green for var. *grubii* and orange for var. *neoformans*), the coordinates of the serotype-specific genes were randomly projected 1,000 times with *BEDTools* version 2.25.0, and the number of overlaps with the chromosome terminal regions was evaluated for each randomization[45]. Considering the null hypothesis that variety-specific genes are randomly distributed in the genome, the alternative hypothesis that the variety-specific genes are concentrated on the chromosome extremities has an empirical *P*-value of 0.36 for var. *neoformans*, and <0.001 for var. *grubii*.

45 Quinlan, A. R. & Hall, I. M. BEDTools: a flexible suite of utilities for comparing genomic features. *Bioinformatics* **26**, 841-842, doi:10.1093/bioinformatics/btq033 (2010).

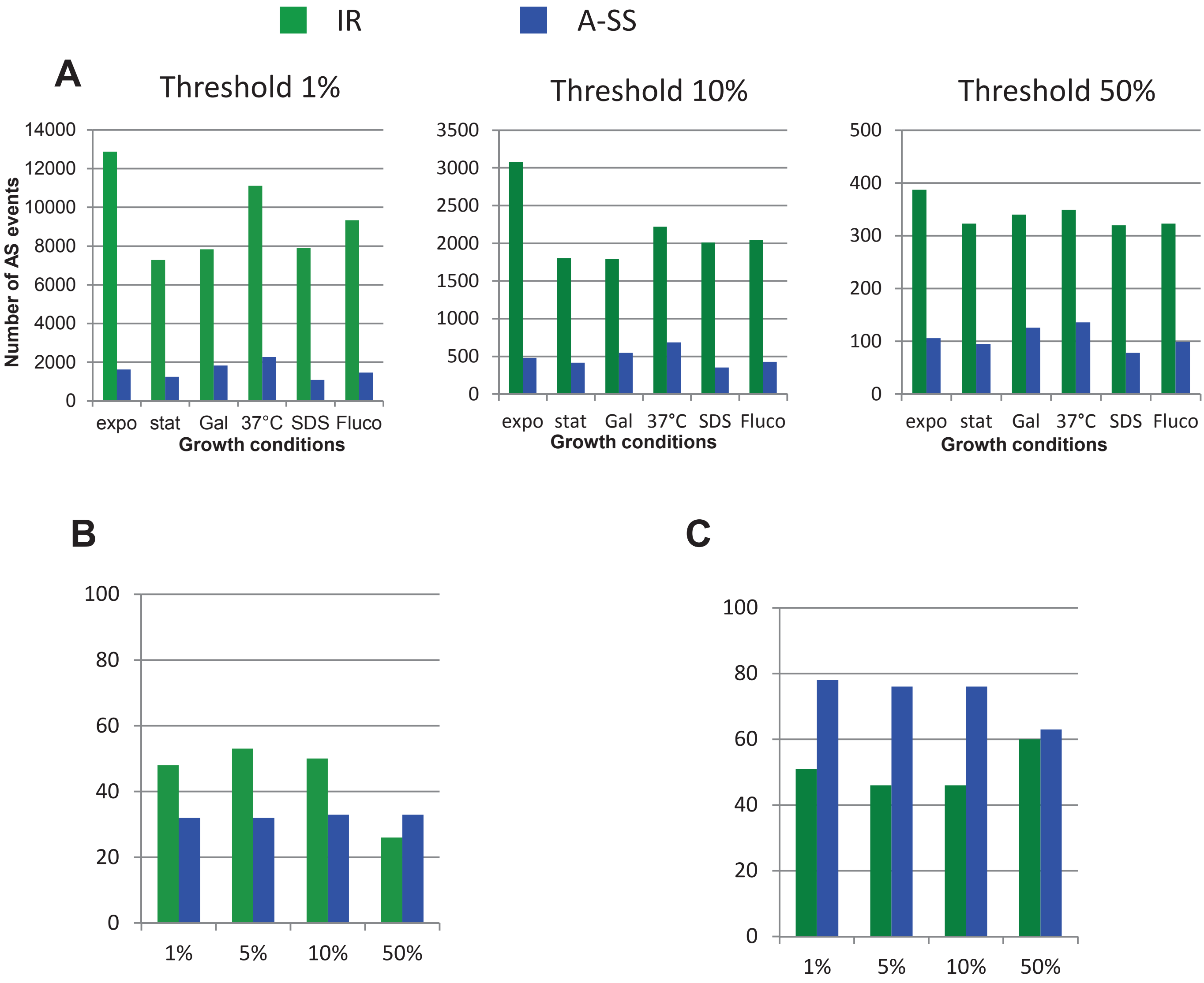

Figure S2. (A) Influence of the choice of the threshold on the number of alternative splicing events identified in each condition. (B) Influence of the choice of the threshold on the percentage of alternative splicing events identified as regulated by growth conditions (C) Influence of the threshold on the percentage regulated alternative splicing events identified as specifically regulated by one modification of the environment.

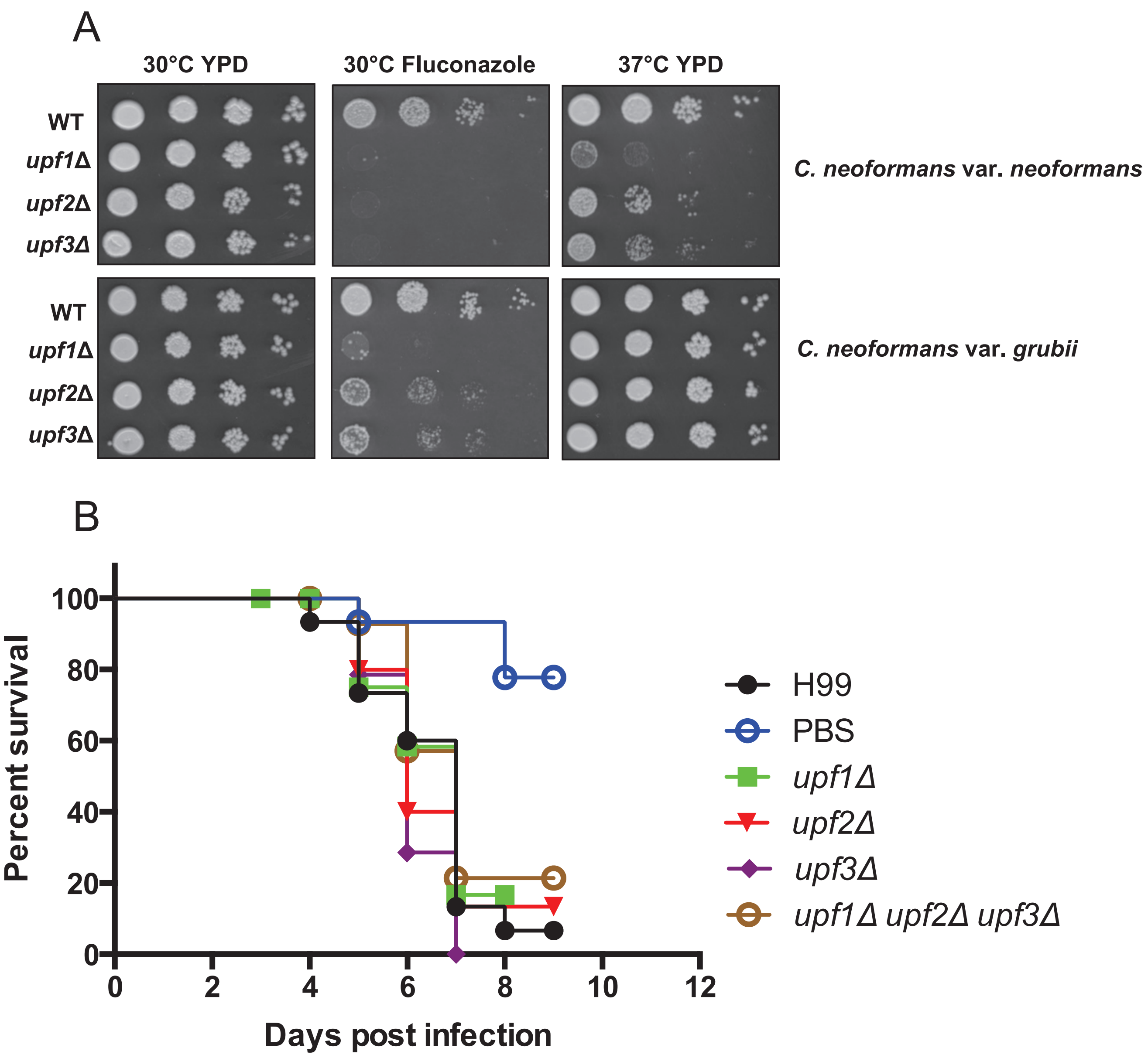

Figure S3. **Phenotypes of the *C. neoformans upf* mutant strains. (A)** Growth phenotypes of the NMD mutant strains. Serial dilutions of cells were spotted on YPD solid medium alone or with fluconazole (14 µg/mL). Pictures were taken after 3 days of incubation. **(B)** Virulence of the NMD mutant strains. The wild-type (H99S) and the *upf1*Δ (YSB2473), *upf2*Δ (YSB2467), *upf3*Δ (YSB2818), and *upf1*Δ *upf2*Δ *upf3*Δ (YSB878) mutants were grown for 16 hours in liquid YPD medium, washed 3 times with PBS, and inoculated into *Galleria mellonella* of the last larval stage at 4,000 cells/larva (15 larva per group). The infected larvae were incubated at 37°C, and survival was monitored for up to 12 days. Statistical analysis was performed by log-rank (Mantel-Cox) test. Median survival for each strain was as follows: H99S strain: 7 days; *upf1*Δ mutant: 7 days; *upf2*Δ mutants: 6-7 days; *upf3*Δ mutants: 6 days; *upf1*Δ *upf2*Δ *upf3*Δ mutants: 7 days. Survival differences between H99S and each mutant strain were not statistically significant.

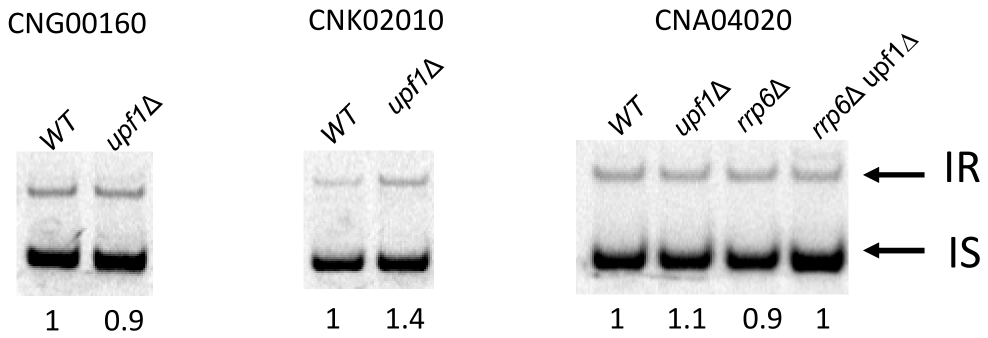

Figure S4. **Analysis of the consequences of *upf1Δ* mutation on intron retention regulation.** Actin mRNA was used as a control. IR and IS stand for intron retention and intron splicing, respectively. The IR/CS ratio was measured in each genetic background and compared to the ratios obtained for the wild-type strain.

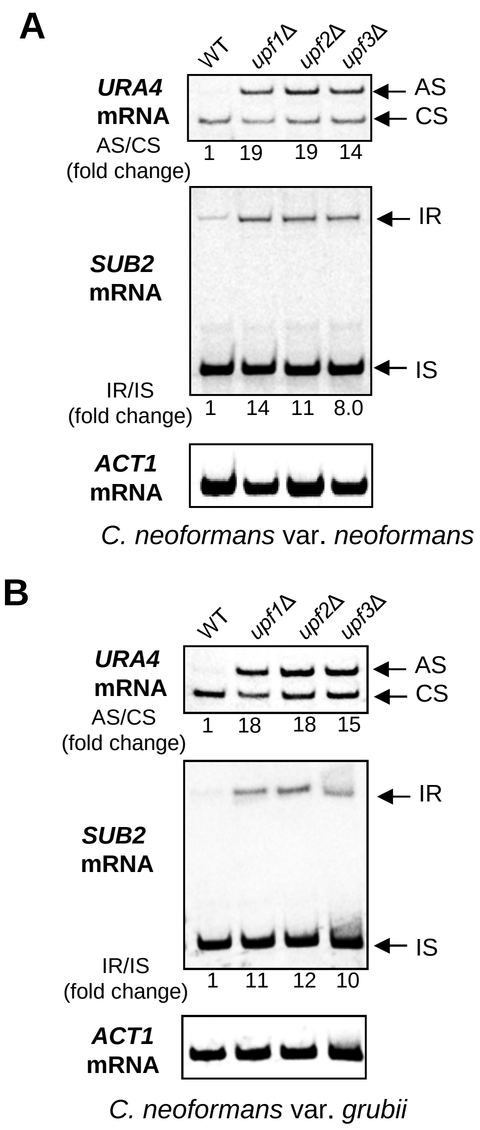

Figure S5: **Effect of mutation of the *UPF* encoding genes on NMD in both *C. neoformans* varieties.** **(A)** RT-PCR analysis of non-productive alternative splice site selection (*URA4*) and intron retention regulation (*SUB2*) in *upf* in *C. neoformans* var. *neoformans* mutant strains. **(B)** RT-PCR analysis of non-productive alternative splice site selection (*URA4;* CNAG_00734) and intron retention regulation (*SUB2;* CNAG_00741) in *C. neoformans* var. *grubii upf* mutant strains. Actin mRNA was used as a control. CS, AS, IR, and IS stand for constitutive splicing, alternative splicing, intron retention and intron splicing, respectively. The IR/IS and AS/CS ratios were measured in each genetic background and compared to the ratios obtained for the wild-type strain.

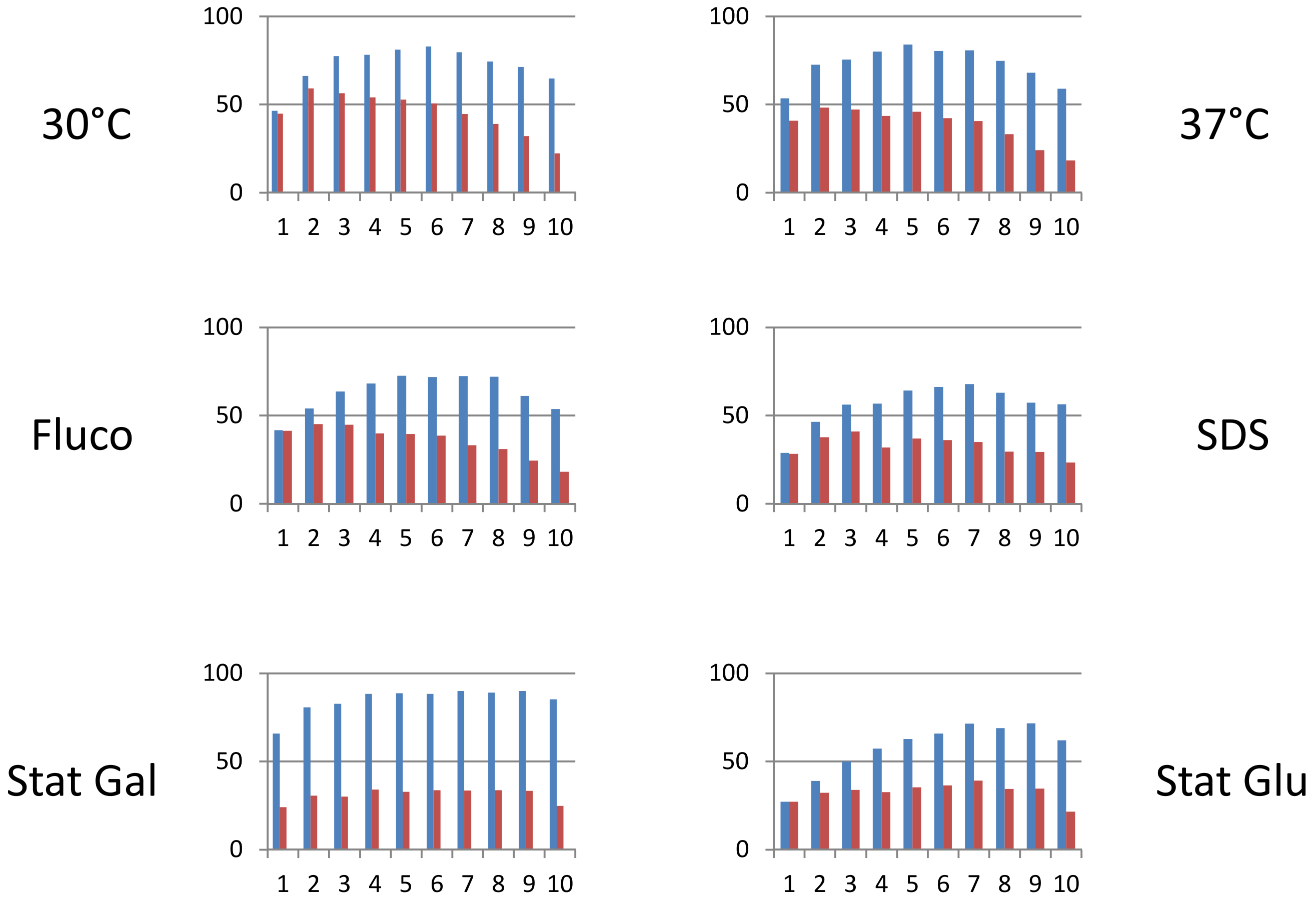

Figure S6: **Percentage of genes regulated by IR** by decile. Genes were classified in deciles according to their level of expression in each condition. The first decile encompasses genes with the lowest expression levels. For each condition, the percentage of genes regulated by IR (1% threshold blue; 5% threshold red) is reported.

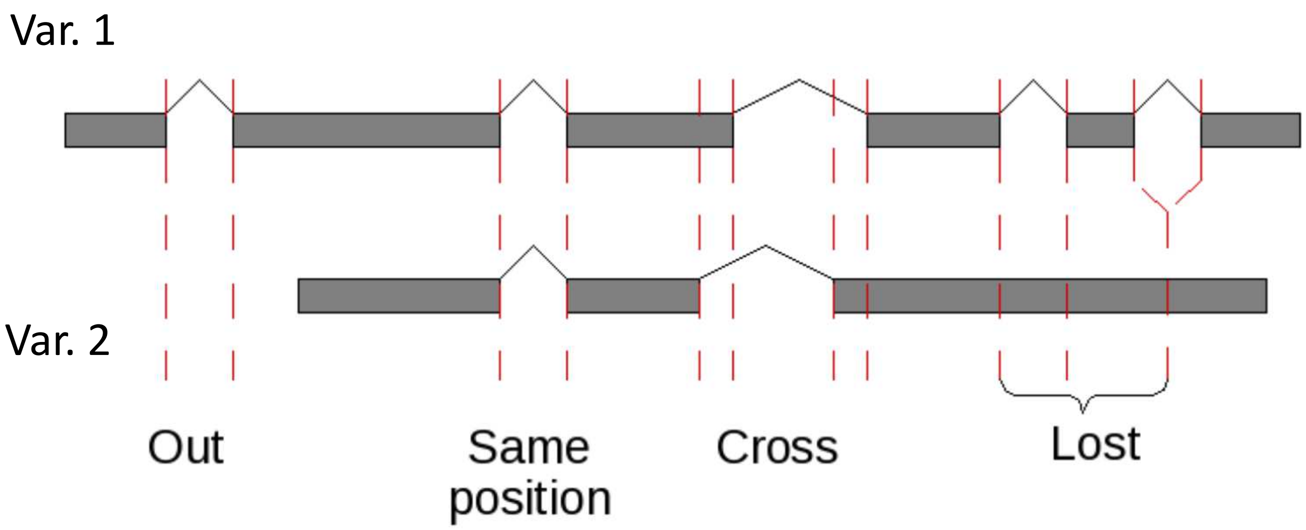

Figure S7: **Method of intron position comparison between the two *C. neoformans* varieties.** Introns from each variety were defined as conserved, lost, cross, or out according to its presence or position in the other variety.

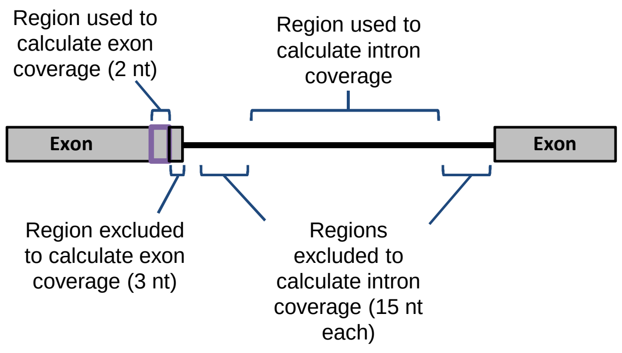

Figure S8: **Schematic representation of the strategy used to measure intron retention.** Expression within the intron was compared with the expression within the upstream exon. A 2-nt window upstream of the intron was considered to exclude 3 nt. Similarly, 2 intronic 15-nt exclusion areas were not considered for these measurements.

Supplementary Figure S9. Full-length blots/gels are presented below

Figure 2C

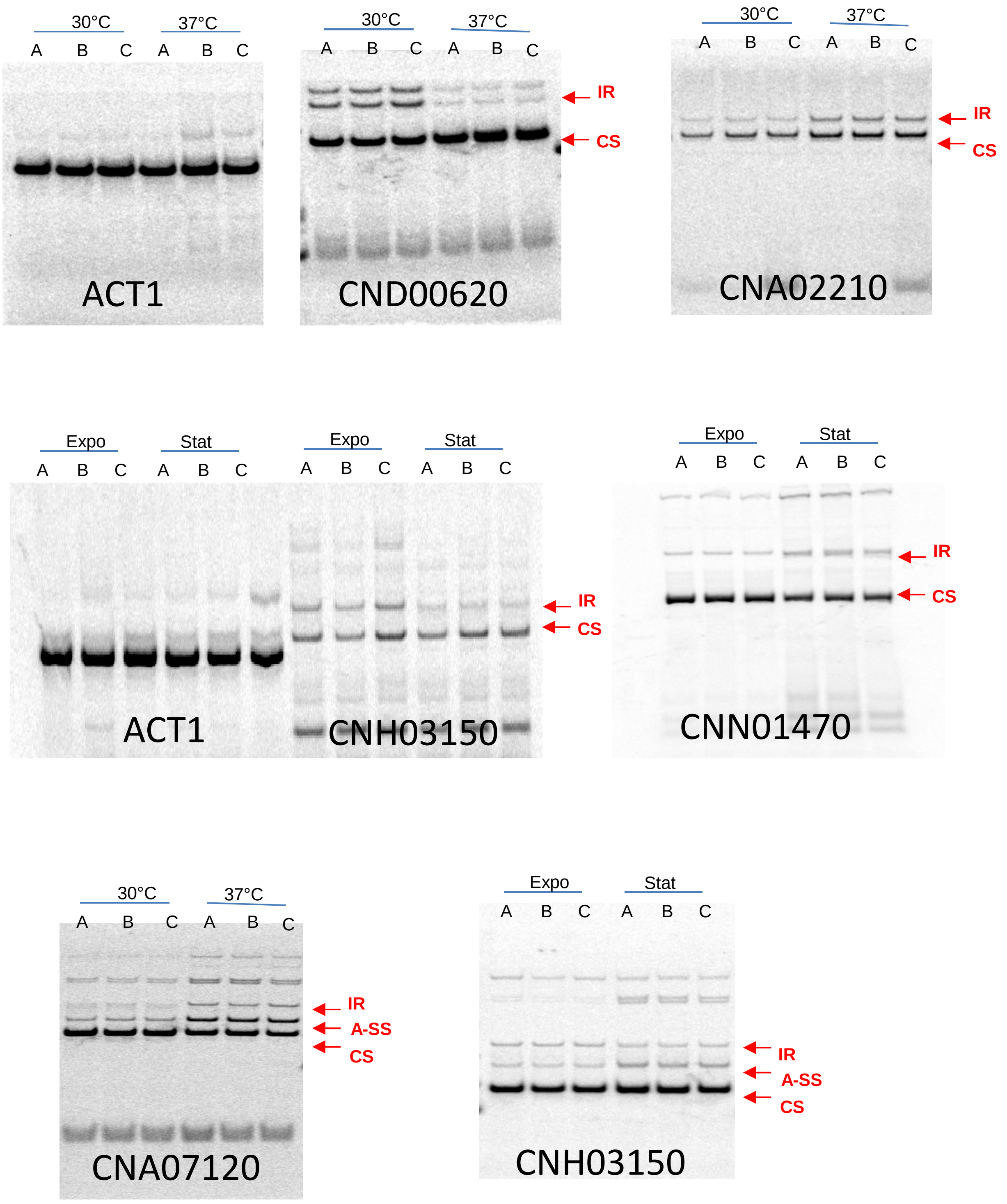
30°C
37°C
A B C A B C
ACT1
CND00620
IR
CS
CNA02210
Expo
Stat
CNH03150
CNN01470
CNA07120
A-SS

Figure 4A

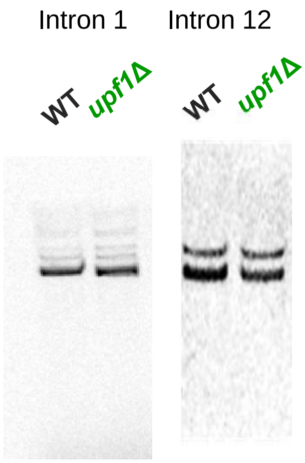

Figure 4B

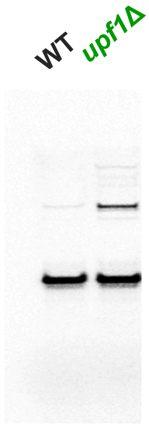

Figure 4C

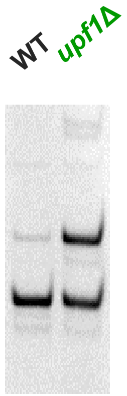

Figure 4D

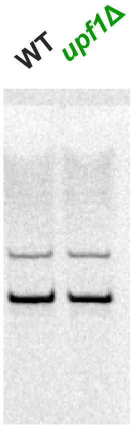

Figure 6A

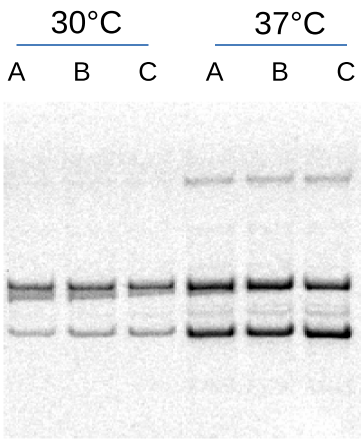

Supplement: supplemtary figures and tables [file srep32252-s1.pdf]
